# Scraps

**Published:** 1891-03

**Authors:** 


### SCRAPS

PICKED UP BY THE ASSISTANT-EDITOR.

---

**L. S. D.**—A subscriber to the *Journal* says that, considering the large incomes made by the dentists, the initials so often appearing after their names are given in the wrong order.

**Pretty Good for a Six Year Old.**—Dr. A. H. S. writes: I circumcised a little fellow six years of age the other day, and when he recovered from the anæsthetic he remarked, "That is the meanest thing one fellow ever did to another."—*The Medical Record.*

**Helping the Doctor.**—A country editor lay in an unconscious condition, and for some time it was feared that he was dead. "Can't you rouse him, doctor?" was anxiously asked. "No," the man of medicine replied. "I fear that life is extinct." Then the editor's assistant bent over and whispered in his ear, "A gentleman wants to put an advertisement in the paper." Immediately the unconscious man's face showed signs of returning life, and, struggling to a sitting posture, he said feebly, "How many lines?"

**The Dancing Mania.**—The effects of the Black Death (see *Journal*, September, 1889) had not yet subsided, and the graves of millions of its victims were scarcely closed, when a strange delusion arose in Germany, which took possession of the minds of men, and, in spite of the divinity of our nature, hurried away body and soul into the magic circle of hellish superstition. An old German Chronicle says:

> Viel hundert fingen zu Strassburg an
> Zu tanzen und springen Frau und Mann,
> Am offnen Markt, Gassen und Strassen
> Tag und Nacht ihrer viel nicht assen.
> Bis ihn das Wüthen wieder gelag.
> St. Vits Tanz ward genannt die Plag.

St. Vitus, whose name became associated with this affection, was a Sicilian youth who suffered martyrdom at the time of the persecution of the Christians, under Diocletian, in the year 303. In course of time he was ranked among the fourteen saintly helpers, and about the end of the fourteenth century a legend was invented that just before he bent his neck to the sword, he prayed to God that he might protect from the Dancing Mania all those who should solemnise the day of his commemoration and fast upon its eve, and that thereupon a voice from heaven was heard, saying, "Vitus, thy prayer is accepted." Thus St. Vitus became the patron saint of those afflicted with the dancing plague, as St. Martin, of Tours, was at one time the succourer of persons in small-pox; St. Antonius of those suffering under the "hellish fire," and as St. Margaret was the Juno Lucina of puerperal women. It was not until the beginning of the sixteenth century that St. Vitus's Dance, of the demoniacal origin of which no one had hitherto entertained the least doubt, was made the subject of medical research. This must be credited to Paracelsus, that mighty, but as yet scarcely comprehended, reformer of medicine, whose aim it was to withdraw diseases from the pale of miraculous interposition and saintly influences, and explain their causes upon principles deduced from his knowledge of the human frame. From his time, the affection became every year more rare, so that at the beginning of the seventeenth century it was observed only occasionally in its ancient form.—Hecker's *Epidemics of the Middle Ages.*

Chorea, which in popular and professional language has the term St. Vitus's Dance as a synonym, has appeared in more recent times as an epidemic, and, as is well known, occasionally spreads by imitation; but it is a very different thing from the complaint described above.

"**A meere dull Phiſitian.**"—In 1628 Edward Blount, the publisher of the 1623 Folio of Shakspere's plays, "aduentur'd to play the Mid-wifes part, helping to bring forth theſe Infants into the World, which the Father would haue ſmoothered: who hauing left them lapt vp in looſe Sheets, as ſoon as his Fancy was deliuered of them," and so gathered into a volume entitled *Micro-coſmographie* the "Essayes and Characters" which John Earle, afterwards Bishop of Worcester and then of Salisbury, had "written eſpecially for his priuate Recreation." The following is his "Character" of a physician of the period:—

His practice is ſome buſineſſe at bed-ſides, and his ſpeculation an Vrinall. Hee is diſtinguiſht from an Empericke by a round veluet cap, and Doctors gowne, yet no man takes degrees more ſuperfluouſly, for he is Doctor howſoeuer. He is ſworne to *Galen* and *Hypocrates*, as Vniuerſity men to their ſtatues, though they neuer ſaw them, and his diſcourſe is all Aphoriſmes, though his reading be onely *Alexis* of Piemont, or the Regiment of Health. The beſt Cure he ha's done is vpon his own purſe, which from a leane ſickelineſſe he hath made luſty, and in fleſh. His learning conſiſts much in reckoning vp the hard names of diſeaſes, and the ſuperſcriptions of Gallypots in his Apothecaries Shoppe, which are rank't in his Shelues, and the Doctors memory. He is indeed only languag'd in diſeaſes, and ſpeakes Greeke many times when he knows not. If he haue beene but a by-ſtander at some deſperate recouery, he is ſlandered with it, though he be guiltleſſe; and this breeds his reputation, and that his Practice; for his skill is meerly opinion. Of al odors he likes beſt the ſmel of Vrine, and holds *Veſpatians* rule, that no gaine is vnſauory. If you ſend this once to him, you muſt reſolue to be ſicke howſoeuer, for he will neuer leaue examining your Water till hee haue ſhakt it into a diſeaſe. Then follows a writ to his drugger in a ſtrange tongue, which hee vnderſtands though he cannot conſter. If he ſee you himſelfe, his preſence is the worſt viſitation: for if he cannot heale your ſicknes, he will bee ſure to helpe it. Hee tranſlates his Apothecaries Shop into your Chamber, and the very Windowes and benches muſt take Phiſicke. He tels you your Maladie in Greeke, though it be but a cold or head ach: which by good endeauour and diligence he may bring to ſome moment indeed; his moſt vnfaithfull act is, that hee leaues a man gaſping, and his pretence is, death and he haue a quarrell, and muſt not meet; but his feare is, leaſt the Carcaſſe ſhould bleed. Anotomies and other ſpectacles of Mortalitie haue hardened him, and hee's no more ſtruck with a Funerall than a Grauemaker. Noblemen vſe him for a director of their ſtomacks, and Ladies for wantonneſſe, eſpecially if hee bee a proper man. If he be ſingle, he is in league with his Shee-Apothecary, and becauſe it is the Phyſitian, the huſband is Patient. If he haue leaſure to be idle (that is to ſtudy) he ha's a ſmatch at Alcumy, and is ſicke of the Philoſophers ſtone, a diſeaſe vncurable, but by an abundant Phlebotomy of the purſe. His two maine oppoſites are a Mountebanke and a good Woman, and hee neuer ſhewes his learning ſo much as in an inuectiue againſt them, and their boxes. In concluſion he is a ſucking conſumption, and a very brother to the wormes, for they are both ingendred out of mans corruption.

